# A Strength-Based Intervention to Increase Participation in Leisure Activities in Children with Neuropsychiatric Disabilities: A Pilot Study

**DOI:** 10.1155/2020/1358707

**Published:** 2020-04-02

**Authors:** Anna Ullenhag, Mats Granlund, Lena Almqvist, Lena Krumlinde-Sundholm

**Affiliations:** ^1^Department of Women's and Children's Health, Karolinska Institute, Solnavägen 1, 171 77 Solna, Stockholm, Sweden; ^2^Academy Health, Social Care and Welfare, Mälardalens University, Box 883, 721 23 Västerås, Sweden; ^3^CHILD, SIDR, School of Health and Welfare, Jönköping University, Box 1026 Jönköping, Sweden

## Abstract

The aim is to evaluate the feasibility of an intervention model with a client-centered goal-directed approach with the aim to enhance the child's participation in leisure activities, self-efficacy, and activity performance. A pilot intervention using a client-centered goal-directed approach and a single-subject design was performed. Two Swedish boys with neuropsychiatric diagnosis aged 12 and 14 years old were included, and 3 leisure activity goals were identified. The intervention was carried out over 8 weeks and took place in the adolescent's everyday environment and at the pediatric rehabilitation center. The goal attainment of participation goals (GAS), the perceived performance ability according to the Canadian Occupational Performance Measure (COPM), the self-efficacy, and the participants' satisfaction were used to study the effect. The participants succeeded in attaining their leisure goals as specified by the GAS by achieving +2 on one goal and +1 on the other two goals. They estimated higher performance ability and self-efficacy in their goal performance. Participants, parents, and therapists were overall satisfied and found the intervention to be applicable and helpful in optimizing leisure participation. The intervention model with a client-centered goal-directed approach in which participants define their own leisure activity goals appears to be effective in increasing participation in leisure activities.

## 1. Introduction

The opportunity to participate and be involved in leisure activities is essential for health and well-being [1]. Participation in leisure activities has positive effects, such as providing children with a sense of belonging and opportunities to fulfill personal goals that may develop their personality [1–3].

Children with neurodevelopmental disorders (NDD) such as autism spectrum disorder (ASD) or children with attention-deficit hyperactivity disorder (ADHD) are reported to experience participation restrictions in leisure activities [4–6]. The restrictions probably are dependent on several influences both internal and external to the child. Common internal influences in children with high-functioning ASD are stereotyped patterns of behavior and impairments in social and occupational functioning. These impairments might particularly have a negative impact on leisure activities, of a social and/or more spontaneous character [7]. Children with ADHD have problems with attention and can fail to inhibit their emotional reactions. Such internal influences probably interact with how peers react to these children, and negative social interaction spirals can be enhanced [8]. Most research on participation restrictions in leisure activities has been focused on children with physical disabilities [9–12], and therefore, intervention studies focusing on participation in leisure activities with peers of children with ASD or ADHD activities are needed.

Imms et al. have conceptualized participation in the Family of Participation-Related Construct (fPRC) framework and describe participation as having two dimensions: *attendance* and *involvement* [13]. Attendance includes the diversity and frequency of performing activities, while involvement focuses on the experience while attending, such as degree of engagement and motivation while attending the activity [13]. The fPRC framework considers activity competence, the sense of self, and preferences as personal intrinsic factors that are influencing and are influenced by participation. Activity competence will affect what skills a child can perform independently in an activity. When children can perform and are highly involved in an activity, they experience a sense of mastery and increased self-efficacy, i.e., one aspect of their sense of self [14, 15] that probably strengthens participatory experiences [15–17]. The child's preferences and intrinsic motivation both are influenced by and influence leisure participation in which children experience activity competence and self-efficacy [18–20]. Child-determined goals for interventions, based on their activity competence, self-efficacy, and interests, need to be formulated [16, 21, 22].

To set child-determined goals for participation interventions, children need to respond to questions about their own participation [23–25]. Still, few studies capture children's opinions directly, and the use of parents or therapists as proxies is common [24, 26]. A means to gather information about children's patterns of participation is to use self-ratings [27, 28]. One instrument that can be used for both self-ratings and proxy ratings is the Children's Assessment of Participation and Enjoyment/Preferences for Activities of Children (CAPE/PAC) aiming to describe children's perception of participation in leisure activities and recreation [29]. Child-determined goals can be identified by using CAPE/PAC [30].

Interventions using individually tailored education and mentoring programs have been found to enhance participation in children with disabilities [31]. It is not clear if these interventions have used a client-centered approach based on child-determined goals. Several studies have demonstrated that children and families who are actively involved in the process of the rehabilitation have better rehabilitation outcomes [17]. Peer discussion groups have also shown to enhance the social participation of children with autism spectrum disorders [32].

The aim of this pilot study was to evaluate the feasibility of an intervention model with a client-centered goal-directed approach. Based on the fPRC model, we used CAPE/PAC to identify children's goals for participation in leisure activities (preferences) and measure self-efficacy (sense of self) and the performance ability (self-rated activity competence). A single-subject design was implemented to adapt the pilot study to a typical clinical setting.

## 2. Materials and Methods

### 2.1. Design

A pilot study with a single-subject AB design across subjects' perceived performance ability, satisfaction with performance, and self-efficacy, as well as actual goal attainment, was used to study the effect of an intervention. The goal attainment approach was chosen to strengthen the conceptualization and delivery of the intervention and to enhance participants' motivation.

### 2.2. Participants

The group targeted for the intervention included children with NDD diagnoses, i.e., ASD and ADHD. Further inclusion criteria were (1) age 11 to 14 years; (2) no, or only mild, intellectual impairment; and (3) a family or social network that had expressed a commitment to support the child through the intervention. The reason for only including children with no or mild intellectual impairment was to facilitate that all participants could participate in the assessments, identify important goals of intervention, and participate in group discussions.

A convenience sample of 10 children, receiving services from pediatric rehabilitation services in an urban area in Sweden, was invited to participate by their therapists verbally and in writing. Seven children/families were not interested to participate due to lack of time or due to other planned treatments. Three of the invited children, all boys with Swedish ethnicity, attended the intervention after written consent from both parents and children. One of the boys dropped out after three weeks due to extra tutoring at school, and thus, only two children remained.

The two participants were John (pseudonym) who was 12 years old with a diagnosis of ASD and ADHD and Charlie (pseudonym), 14 years old with high-functioning autism spectrum disorder. Both of the boys lived with their families in a city in Sweden. Before the intervention, John had complained about not having any organized leisure activities to attain to and Charlie's mother had expressed a wish that her son should improve his social skills.

Two therapists working at the pediatric rehabilitation center, an occupational therapist, and a youth worker participated, together with a physiotherapist (A.U.) who also was a member of the research team that delivered the intervention. All of the therapists had over 10 years experience of working in pediatric rehabilitation centers for children in need of special support.

### 2.3. Intervention

The intervention consisted of individual training at home or in the community and group meetings at the pediatric rehabilitation center. Following a client-centered goal setting process and creation of a goal scale, the intervention was implemented over eight weeks, including seven group meetings ones a week, each of duration 1.5 hours. To involve the child in the process, the therapist strived to enable the child's sense of control over the intervention and ensured that strategies for goal attainment were feasible [33]. This was done by weekly discussions and evaluations of goals and strategies together with the children.

In the goal setting process, the children's responses to the CAPE and PAC were used to find preferred goal activities of the children, and the Goal Attainment Scaling (GAS) was used to formulate levels of goal achievement according to the children's chosen goals.

During the eight weeks of intervention, the child worked with different strategies to attain their individual goals, together with the therapist, the parent, or alone, either at home or in the setting in which the activity was taking place (see Table 1, summary of strategies and intervention provided). For example, the child could practice coordination skills required for inline skating together with the therapist in the street at home. To tailor the treatment to individual goals, the therapists needed competences of motor learning, adaptive function, how to amend the environment and obtain information of community recreation, and leisure activities [34, 35].

The chosen strategies and the duration and frequency of practice were recorded once a week during the group meetings at the pediatric rehabilitation center by the therapist and by the child using logbooks. The child also logged the experienced enjoyment of the use of strategies during the intervention. The logbooks were used to help the therapists and the children to know what to do, where, when, and how often. With the logbooks, it was also possible for the therapists to follow up on the participants' compliance with the intervention.

The weekly follow-up during the group meetings at the pediatric rehabilitation center was seen as a component of the intervention. It was considered important to continuously evaluate the implemented strategies and identify suitable solutions for goal attainment and to give the child feedback about the implemented strategies and the progress. The work with attaining the participation goals was discussed by the children and the therapists, and estimations of perceived performance ability (COPM) and self-efficacy were done weekly by the child. These estimations formed a basis for the evaluation of the implemented strategies and planning effort.

During the weekly group sessions, the children were encouraged to record one positive and one negative experience concerning the interventions carried out for goal attainment during the past week. It was regarded as an important part of the intervention to stimulate the children's autonomy and self-reflection of their performance and to see the children as competent with abilities to find solutions to problems [21, 33]. The therapists and the children discussed these issues/topics, and the children gave and received feedback on the strategies that had been used. Furthermore, 20 minutes of the meetings were used for discussing and exercising topics such as “How should a good friend be?,” “How can I get in contact with a peer?,” and “What can I do together with a peer?” The group meetings ended with 30 minutes of video gaming to stimulate interaction between the participants and to reward them for their contribution to group discussions.

### 2.4. Assessment Instruments and Outcome Measures

The *Children's Assessment of Participation and Enjoyment/Preferences for Activities of Children (CAPE/PAC)* was used to assess participation. It is appropriate for children and young people with and without disabilities between the ages of 6 and 21 years [29]. The CAPE contains 55 items reporting participation regarding objective aspects (diversity and intensity of participation), contextual aspects (where and with whom activities take place), and subjective aspects (enjoyment) [29]. The PAC is a parallel measure of the child's preferences for activities and includes the same 55 leisure activities as the CAPE. The child answers questions about how much he or she would like to do an activity. Answers are (1) I would not like to do it at all, (2) I would sort of like to do it, and (3) I would really like to do it [29]. The outcome of the CAPE/PAC has demonstrated evidence for reliability and validity in several countries, including Sweden [36–39].

The *Goal Attainment Scaling (GAS)* is a method for individual goal setting and a structured way of recording goal achievement, first introduced by Kiresuk and Sherman in 1968 [40, 41]. Goal Attainment Scaling has been recommended in the literature as a means for enhancing transparency and coordination in goal setting with the child and the family during a child's rehabilitation process [41]. The goal should be Specific, Measurable, Acceptable, Relevant and Time-related (SMART) [42, 43].

The *Canadian Occupational Performance Measure (COPM)* [44] is based on a client-centered approach for the purpose of identifying treatment goals, assessing changes, and giving satisfaction in performance over time. The COPM measures changes in self-care and productive and leisure activities. Evidence for test-retest and interrater reliability has been reported for COPM, and acceptable content, criterion, and construct validity have been demonstrated [45]. In this study, only the scale of the perception of performance ability was used, and for each goal, the child completed a self-evaluation of his or her current performance from 1 (not able to do the activity/goal at all) to 10 (able to do the goal/activity extremely well). The rationale for using the performance ability scale was to obtain a measure of performance that was suitable for multiple repeated measuring and to compare the change of GAS levels with the child's perception of goal performance.

Based on the hypothesis that a child may enhance his or her self-efficacy when attaining a goal, i.e., the sense of self component in the fPRC framework, we believed that it was important to estimate self-efficacy. Since no suitable measure of self-efficacy was found, the children estimated their self-efficacy in coping with the goal activity and finally attaining the goals on a simple rating scale with five statements: 1 (I am not able to attain the goal), 2 (I am limited in my ability to attain the goal), 3 (I am able to attain the goal), 4 (I am quite able to attain the goal), and 5 (I am without doubt very able to attain the goal).

Since this was a pilot trial of an intervention model, it was important not only to analyze the efficacy of the intervention but also to investigate the children's, parents', and therapists' views, satisfaction, and experiences of the intervention model. Therefore, children, parents, and therapists responded to a questionnaire about these topics. The participants furthermore answered questions about the interventions' efficacy and used an ordinal rating scale from 0 to 5 where 0 = not at all useful/efficient and 5 = very much useful/efficient.

Therapists were also asked to rate the feasibility and utility of the CAPE/PAC in measuring participation in leisure activities and to identify the preferred leisure activities of children. A visual analog scale (VAS) from 0 to 10 was utilized where 0 = CAPE/PAC is not at all useful to 10 = CAPE/PAC is very useful. The reason why the participants used a Likert scale instead of VAS was that it was assumed to be easier to understand.

They also answered questions about the efficacy of using GAS, estimation of performance ability (COPM) and self-efficacy, and the logbooks and group meetings.

### 2.5. Ethical Consideration

The Regional Committee for Medical Research Ethics, in Stockholm, Sweden, approved the study of Swedish participants (reference number 2011/823-31/5).

The families received written and verbal information about the study, and written consent from both parents and children were requested.

### 2.6. Procedure

Before the intervention started, the therapists received education and information during two days about barriers and facilitators of participation and of how to stimulate participation in leisure activities. They were also educated on how to administer Goal Attainment Scaling (GAS), Children's Assessment of Participation and Enjoyment/Preferences for Activities of Children (CAPE/PAC), and the perceived ability performance scale of the Canadian Occupational Performance Measure (COPM). The education and information were given by the research team.

The three families who had accepted the invitation attended an evening meeting and received more detailed information about the intervention model and procedure, as well as about the study, and both children and parents had opportunities to raise questions.

**Figure 1:**
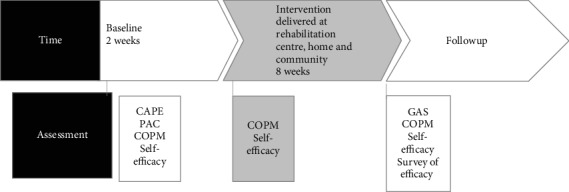
Timeline of assessments, intervention delivery, and follow-up.

The assessment procedure included the following. Firstly, CAPE/PAC data was collected for descriptive and goal setting purposes. The children responded to the CAPE and PAC together with the therapist at the pediatric rehabilitation center. The CAPE was used to get an understanding of the children's patterns of participation, and the children identified their own preferred goal activities by using the PAC. The therapists used the GAS to formulate levels of goal achievement according to the children's chosen goals that the children had to approve. To identify appropriate treatment strategies for goal achievement, the therapists did a careful inventory of the child's abilities such as activity competence, preferences, and possible barriers posed by the environment. Thereafter, repeated baseline measures were conducted together with the children twice a week with the perceived performance ability scale of COPM and self-efficacy estimations for the chosen goals. These data were collected all together for 10 weeks (two weeks baseline and eight weeks of intervention) by the therapist together with the child at the pediatric rehabilitation center. All goals showed a stable baseline after two weeks which allowed the intervention to be introduced. At the end of week 10, all assessments were repeated to evaluate the intervention outcome. The GAS scales were then evaluated by an independent therapist, not involved in the treatment or the study procedure. After the intervention period, at follow-up, families and therapists answered the questionnaires concerning the feasibility of the intervention model and the assessments (see the timeline of assessment, intervention, and follow-up in Figure 1 and the Supplement of a more detailed description of the implemented intervention (available here)).

### 2.7. Analysis

The repeated measures were plotted. The authors independently determined, by visual inspection, if the patterns of results supported the conclusion that the interventions had the hypothesized effect. The visual analysis was based on components such as level, variability, trend, and slope [46]. By visual inspection, patterns in baseline data were compared to those in intervention data to determine the effect of the intervention. Descriptive statistics were used for the remaining data.

## 3. Results

### 3.1. Baseline

**Figure 2:**
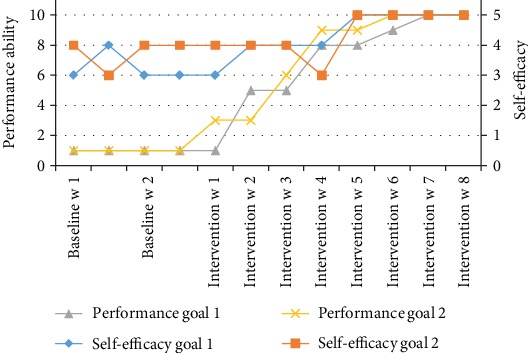
John's estimations of performance ability and self-efficacy of goal 1 = inlines and goal 2 = jujutsu.

**Figure 3:**
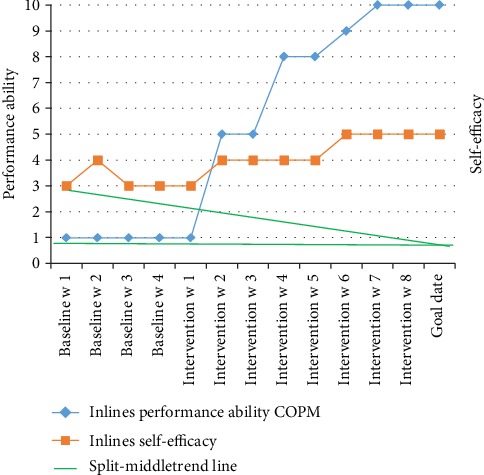
Visual inspection split-middle trend line for goal 1 = inlines.

**Figure 4:**
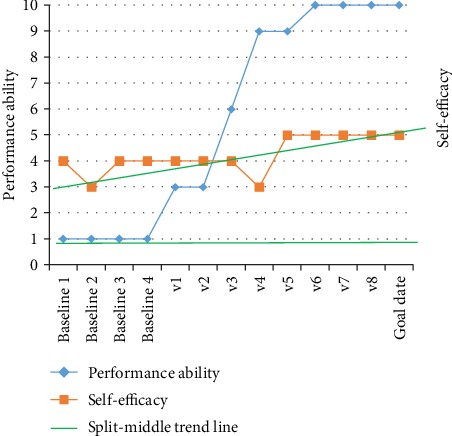
Visual inspection split-middle trend line for goal 2 = jujutsu.

The CAPE result showed that at baseline, John participated with high diversity but low frequency in social activities. He performed activities such as talking on the phone, going to a movie, and listening to music. Most of the activities, except for talking on the phone, which he did several times a week, were performed once a month together with his family or relatives. None of the activities were done together with friends. His enjoyment ratings were highest for recreational activities: playing on the computer, playing on the equipment, and pretend play. Regarding preferences for activities (PAC scores), he had the highest preferences for physical activities. Consequently, he chose physical activity goals: inline skating and jujutsu (see Table 2). The estimations of performance ability regarding both inline skating and jujutsu were 1 (I'm not able to attain the goal), and the estimation of self-efficacy concerning inline skating was 3 (I am able to attain the goal) while that of jujutsu was 4 (I am quite able to attain the goal) (Figures 2–4).

**Figure 5:**
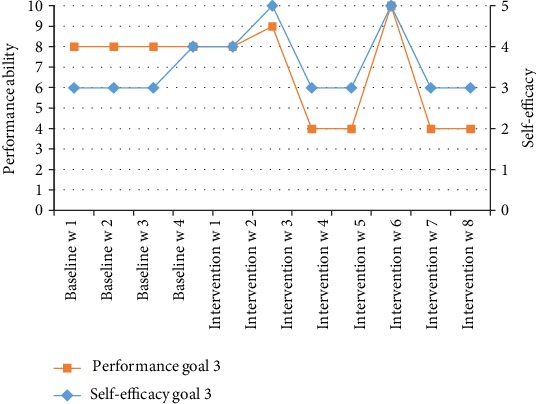
Charlie's estimations of performance ability and self-efficacy of goal 3.

At baseline, the other boy, Charlie, also participated mostly in social activities, talking on the phone, listening to music, making food, going to the movie, going on a full-day outing, etc., followed by recreational activities. All the social and recreational activities were done together with his family or alone, except for playing cards and playing on the computer which were done together with friends or others. He typically enjoyed participating in skill-based and self-improvement activities, religious activity, going to the library, reading, and doing chores, and he had the highest preferences (PAC scores) for social activities, followed by self-improvement activities. Hence, his goal activity was to have a pen pal (see Table 2). At baseline, his estimation of performance ability was 8, and the estimation of self-efficacy was 3 (I am able to attain the goal) for the goal activity (Figure 5).

### 3.2. Outcomes after Intervention

The results of the repeated measures of the performance ability scale and the self-efficacy rating are plotted in Figures 2–4 for the goals of John (goal 1 = inlines and goal 2 = jujutsu) and in Figure 5 (goal 3 = pen pal) for Charlie. Visual inspection showed a pattern of baseline ratings being clearly lower for performance ability and self-efficacy than the ratings during the intervention phase for goals 1 and 2, a judgment shared by all raters. For goal 3, the ratings were highly varying. The performance and self-efficacy ratings during the intervention period increased over time and reached the highest level for goals 1, 2, and 3. The visual inspection indicated a change in level of performance between the baseline and the intervention period for goals 1 and 2 but not for goal 3. The trends and slopes were changed from no slope (horizontal line) in the baseline to a slope of increased performance ability and self-efficacy in the intervention phase regarding goals 1 and 2. For goal 3, the trend and slope were not interpretable because of variability in data (see Figures 2–5).

After the intervention, the two boys had succeeded in attaining their goals as specified by the GAS bypassing the 0 level and even achieving +2 on one goal and +1 on the other two goals (see Table 2).

The results from the questionnaires completed by the two boys and their parents indicated that in their opinion, the intervention had been efficient in improving participation in leisure activities. The two parents particularly appreciated the assessment of leisure activity participation and preferences for leisure activities (CAPE/PAC) and the group meetings (Tables 3 and 4).

The results of the therapists' questionnaire indicated that the occupational therapist and the youth worker found the CAPE and PAC feasible to use in the clinic when working towards increasing participation in leisure activities for children (Table 5). The therapists also appreciated the use of logbooks and the estimations of self-efficacy, and in general, they found the intervention method to be efficient in the clinical work to enhance the pattern of participation in leisure activities. However, they experienced the GAS to be difficult and time-consuming to use, and they thought that the group meetings should be less often. One of the therapists suggested the group meetings would be conducted more frequently at the beginning of the intervention and then carried out every second week of the month.

## 4. Discussion

The aim of this pilot study was to evaluate the feasibility of a client-centered intervention model with a child-determined goal-directed approach. The results indicate that the client-centered intervention for increasing participation in leisure activities was applicable. The children could select and set goals and reached their leisure activity goals. In addition, performance ability and self-efficacy increased over time for two of the three goals. According to the boys and their parents, the intervention had been efficient in improving participation in leisure activities. The therapists found the intervention methods and the CAPE/PAC feasible for clinical use, and they appreciated the use of logbooks.

The focus of this study was to optimize participation in leisure activities with a client-centered approach focusing on the children as clients. Studies have shown that parents sometimes underestimate the child's abilities and have other wishes for the children's participation than children themselves. Therefore, it was essential to use self-ratings of participation to directly seek the children's own preferences and experiences of participation. Self-ratings will also provide children with opportunities to share ideas of desired changes that will give them a sense of control over the intervention [47]. The two children identified preferred leisure activity goals by using the PAC. Preferences are both antecedent to and a consequence of participation, since our past experiences of enjoyment and success when participating will form our future preferences to participate [13].

A survey has indicated that pediatric therapists are not always working on an optimal level regarding screening social, physical, and resource barriers that can hinder their client's participation in leisure activities [48]. In this study, the therapists were encouraged to carefully screen preferences and possible barriers posed by the environment. This required therapeutic competences in motor learning, adaptive function, how to amend the environment, and how to obtain information of community recreation and leisure activities [34, 35, 49]. Besides, to be able to optimize participation in leisure activities, the therapists were required to have parts of their working hours located in the afternoons and the evenings, since that is when leisure activities take place. This dependence on flexible working hours may cause problems with implementation.

The results indicated that GAS was sufficiently sensitive to discriminate changes in task performance following eight weeks of intervention, and the feedback obtained from the scales improved the clarity of the therapy objectives for both therapists and children. Thus, the efficacy of Goal Attainment Scaling as a useful method for measuring outcome and conceptualizing goal-directed intervention was confirmed [30, 42], although practice is needed to successfully use GAS and difficulties to differentiate the goal activity at different levels have been reported [41]. The therapists in this study received education in using GAS but still found it difficult and time-consuming to define goals.

Probably active student engagement involving autonomous goal setting can also lead to higher achievement and higher levels of self-efficacy and self-regulation [50]. The children in this study estimated a higher level of self-efficacy, when they reached their goals at the end of the intervention. Self-efficacy is a judgment about the capability to successfully perform a specific task at a given level. The assumption is that when a child succeeds in attaining an important goal, he or she will experience a sense of control that will reinforce self-efficacy and the sense of self [13–15]. Constructive feedback can strengthen valuable skills and competencies that will reinforce the self-efficacy to handle a certain situation, which, in turn, will stimulate the person to seek new available activities. This can be illustrated by the case of John: when he was given positive feedback and succeeded in attaining his jujutsu activity goal, his estimation of performance ability (COPM) and his self-efficacy increased and he was no longer afraid of occupying a new niche, organized physical activity, and consequently, he began to practice tennis once a week.

The emphasis on a client-centered approach was an essential part of the intervention so that the participating children were involved in the entire process. We believed that they would be more likely to achieve their desired changes/goals if they felt confident and had control of the implementation of the intervention. Partnership, decision-making, and respect have been reported to have positive benefits in client-centered work [33, 51]. The therapist worked closely with the children, and during the weekly group meetings, the therapists enabled the children to analyze their performed strategies and those of others, thereby supporting problem-solving. The therapist interacted with the child and tried to make the child feel competent and active. Logbooks were used to structure the work that was necessary for goal attainment so that the therapists, children, and family knew what to do, when, how often, and by whom. The therapists in particular, but also the children, seemed to appreciate using the logbooks because it helped them to organize the work and “they knew what to do.”

The effect of social training was not examined in this study, but both of the boys perceived that they had increased their knowledge of being together with peers by attending the group meetings. The parents found their boys to have become more socially active, and one of the boys (John) had begun to play with a peer during the school recess. Studies have shown that children with neurological disabilities can benefit from discussion groups of peers and training in social communication skills, which will increase their social participation [4, 32, 52]. In this study, the children discussed how a good friend should be, what to do together with a friend, how to make contact with a peer, presentation of one's strengths/skills/abilities and interests, what to do when I disagree with a friend, etc. The children were active in the group discussions and acted out situations of what to do when disagreeing with a friend and how to make contact with a peer in role-playing. Unfortunately, there were only two participant children in the intervention, and more participants are necessary to achieve a better group.

## 5. Conclusions

The purpose of conducting this pilot study was to examine the feasibility of a client-centered intervention model intended to be used in a lager study. We believed that in this first step, a single case design can provide more detailed insight into both intervention methodology and children's patterns of participation than survey data or RCTs [53]. Research with a larger sample of participants is required to confirm the results of the intervention. Concerning the intervention methods used, our result indicates that GAS might be difficult to use without education and experience, something also previously reported [41]. Therapists found it time-consuming to use GAS, and the goal scales in this study leave room for improvements. Another issue is the intensity/frequency of intervention. The therapists found the intervention to be too time-consuming with both weekly group meetings and individual contacts with the child between the group meetings. The oldest boy participating in the study reported having problems in carrying out the strategies for goal attainment due to long schooldays and homework. A more optimal concept might be to have fewer group meetings, once every second week of the month, for example, and implementing the communication, planning, and evaluation of implemented strategies in a web-based logbook.

Several reflections have arisen from this pilot study which are worthy of further attention. First, children with high-functioning autism spectrum disorders can identify and attain leisure activity goals that are important to them, and the CAPE and PAC can be used as a self-rating tool in this work. Second, the analysis of the child's personal and environmental barriers and facilitators is critical in providing the therapist with ideas of what strategies to be implemented for goal attainment. Finally, a client-centered approach, in which all involved work together respectfully and supportively, can probably enhance goal attainment. The therapists need to learn and apply strategies that motivate the child, create an understanding of the intervention process, and support the child in decision-making.

## Figures and Tables

**Table 1:** Summary of strategies and intervention provided.

Child occupational goals	Intervention strategy	Intervention provided
Individual/child factors		
Jujutsu	Instruction and practice of physical performance of coordination and body control	The therapist demonstrated and instructed step-by-step how John could coordinate arms and legs. Practicing step sequences of jujutsu to be able to coordinate arms, legs, and trunk while doing jujutsu step sequences and the technique of falling backward
	Facilitation of autonomy and mobility skills	Instructions on how to find the bus timetable and encouragement of autonomy by going alone by bus to and from the jujutsu training
	Learning the vocabulary of jujutsu, the most common instructions in Japanese	Finding a jujutsu vocabulary on the internet and learning the most common instructions used in the training lessons

Inline skating	Instruction and practice of physical performance of coordination for inline skating	The therapist demonstrated and instructed how to transfer weight onto one leg and push away with the other to increase the speed and how to coordinate the weight on one leg and transfer the center of gravity in front of the body when stopping
	Facilitation of encouragement in inline skating without holding on	Positive feedback of John's performance skills of balancing with inlines and of his coordination ability of arms and legs. Encouragement to skate without holding on, successively increasing the distance between the therapist and John

Pen pal	Instruction and practice on how to find a website	Strategies on how to search for a website of pen pals on the internet and positive feedback of the strategies used
	Instruction and practice of how to design and write a letter	Discussions of what to write in a letter and how to design the layout of the letter

Environmental factors		
Jujutsu	Consultation with the jujutsu instructor and parents	Discussing and inquiring about John's ability and participation in the jujutsu training. Strategies of direct positive feedback, repeating instructions step-by-step in short sequences. Communication and instruction of parents of how they could help John with practice at home
	Inventory of suitable martial arts clubs	Finding a suitable jujutsu club with experience in handling children with disabilities
	Facilitation of somersaults	Supplying a wedge and providing instructions in how to do somersaults on it

Pen pals	Inventory of cycle paths and facilitation of performance ability of inline skating	The therapist discussed suitable cycle paths for practicing inline with the family and identified more difficult cycle paths, including slopes, after progress in mobility. The therapist sometimes needed to brush the paths to facilitate skating

**Table 2:** Goal Attainment Scaling: the achieved levels are in bold.

Goal level	Jujutsu	Inline skating	Have a pen pal
-2	Has never practiced jujutsu	Has never done inline skating	Has not written a letter to a pen pal
-1	Has actively participated 30 minutes in jujutsu training	Can do inline skating 50 meters and stop without falling	Find a website for pen pals and a person to send a letter to
0	Has actively participated more than 30 minutes 5 times in jujutsu training	Can do inline skating 1000 meters and stop without falling	Write a letter to a pen pal with assistance
+1	Has actively participated more than 30 minutes 5 times in jujutsu training and has once gone there by himself by bus	**Can do inline skating 1000 meters and make turns around cones without falling**	**Write a letter to a pen pal without assistance**
+2	**Has participated regularly once/week (>more than 5 times) and gone by himself by bus to and from jujutsu**	Can do inline skating more than 1000 meters, make turns around cones, and skate down slopes without falling	Write more than two letters and reply to the pen pal

**Table 3:** The children's and families' evaluations of the intervention (1 = not at all agree, 5 = totally agree).

	John	John's parent	Charlie	Charlie's parent
I have/my child has attained the goal	5	5	5	3
I have/my child has obtained a higher self-efficacy	4	4	3	4
I have/my child has increased his participation in leisure activities	5	5	3	4
I have/my child has enjoyed in attending the group meetings	4	5	3	4
I have/my child has learned new skills about being in a group with peers	4	2	4	2
I have/my child has enjoyed carrying out planned activities in the logbook	5	4	2	3
I'm satisfied with the intervention	4	4	4	2
It was important to choose my own goals	4	—	4	—

**Table 4:** Open questions to parents.

What do you appreciate with the intervention?	*“I very much appreciated the evaluation of my son's leisure interests and that he was given the opportunity to decide by himself which leisure activity he would like to participate in” (John's parent)* *“For my son it was positive to meet the therapists. Even though he had a busy term he always wanted to attend the group meetings. I also think he has become more extroverted.” (Charlie's parent)*
Is there anything you missed out?	*“I think the information about the families' engagement needs to be clearer. We thought that the therapists would do all the work.” (John's parent)* *“There were too few participants. My son wanted to meet peers in his own age.” (Charlie's parent)*

**Table 5:** The therapists' evaluation of the intervention (1 = not at all useful, 10 = very useful).

	Occupational therapist	Youth worker
The feasibility of the CAPE to evaluate patterns of participation	8	8
The feasibility of the PAC to evaluate preferences of activities	8	8
The feasibility of GAS to identify goals	4	6
The feasibility of the performance ability scale in COPM	4	—
The feasibility of the estimation of self-efficacy	8	—
The feasibility of the logbooks to facilitate the work for goal attainment	7	8

## Data Availability

The qualitative and quantitative data used to support the findings of this study are included in the article and within the supplementary information file. The individual assessments and logbooks used in the study are restricted by the Regional Committee for Medical Research Ethics, in Stockholm, Sweden Reference number; 2011/823-31/5. Data are available from the corresponding author for researchers who meet the criteria for access to confidential data.

## Supplementary Materials

Supplementary Materials Supplement of the implemented intervention: (1) measurement of participation patterns and preferences of leisure activities, (2) assessment of child-based and environmental facilitators and barriers and strategies of implementation, (3) implementation of strategies for goal attainment, (4) evaluation of the intervention process and outcomes, (5) Charlie (pseudonym), (6) measurement of participation patterns and preferences of leisure activities, (7) assessment of child-based and environmental facilitators and barriers and strategies of implementation, (8) implementation of strategies for goal attainment, and (9) evaluation of the intervention process and outcomes.
