# Supplementary material for: A Strength-Based Intervention to Increase Participation in Leisure Activities in Children with Neuropsychiatric Disabilities: A Pilot Study
Source: Occup Ther Int. 2020 Apr 2;2020:1358707. doi: 10.1155/2020/1358707 (PMC7154983; doi:10.1155/2020/1358707)
Supplement: Supplementary Materials — Supplement of the implemented intervention: (1) measurement of participation patterns and preferences of leisure activities, (2) assessment of child-based and environmental facilitators and barriers and strategies of implementation, (3) implementation of strategies for goal attainment, (4) evaluation of the intervention process and outcomes, (5) Charlie (pseudonym), (6) measurement of participation patterns and preferences of leisure activities, (7) assessment of child-based and environmental facilitators and barriers and strategies of implementation, (8) implementation of strategies for goal attainment, and (9) evaluation of the intervention process and outcomes. [file 1358707.f1.pdf]

Supplement of the implemented intervention

Case report

John (pseudonym)

A physical therapist, who was one of the researchers, implemented the intervention of John. John (pseudonym) is a male, 12 years old with a diagnosis of autism and ADHD. He lives with his family in Sweden. Lately John has complained about not having any organized leisure activities to participate in. His parents state that he has a low self-esteem and is unsure of his ability in leisure activities.

Measurement of participation patterns and preferences of leisure activities

John completed the CAPE and PAC at an interview with a therapist. He participated with the highest diversity in social activities including, talking on the phone, going to party, listening to music, going to movie and going on a full day outing. The second-highest group of diversity was recreational activities. The activities were carried out together with his family or relatives, and recreational activities were performed with the highest frequency.Concerning his preferences for activities, he had the highest preference scores in physical activities. After discussion, John identified two leisure goal activities: one in the martial arts and the other in inline skating. The goals were formulated using GAS, in which -2 represents the current level of performance at baseline, 0 represents the expected outcome and +2 the ideal possible outcome (table 2). At baseline John's estimation of his performance ability for goals 1 and 2 were both 1, and his estimated self-efficacy in coping with the goal activity and attaining the jujutsu and inline goals were 4 and 3 (figure 2-4).

Assessment of child-based and environmental facilitators and barriers, and strategies of implementation

Goal 1

Interviews with John and his family were carried out to obtain knowledge of John's environmental strengths and hindrances. The therapist also observed John during the introductory jujutsu lesson and assisted him during his first try at inline skating. Regarding the goal of jujutsu the assessment revealed that Johns' strengths were: he was interested in attending jujutsu training, he was not afraid of asking the instructor or peers how to do difficult exercises, he was good at observing and trying to imitate the motion sequences of jujutsu and finally he was unafraid to go by bus and had an ability to organize his travel by bus. The identified barriers for participation in jujutsu were: limited body control and

coordination, an inappropriate level of attention with some difficulty in listening to instructions, and the parents' limitation of time to follow him due to full time work and because of their own and the brother's leisure activities, etc.

Goal 2

Concerning the goal of inline skating, John's strengths were: a high motivation, the equipment was available and the neighbourhood contained safe cycle tracks and paths. The identified barriers were a fear of falling and problems with coordination/ transferring weight to one leg, and balancing when he had to stop.

Implementation of strategies for goal attainment

Goal 1

The therapists' roles in the implementation of strategies for goal attainment included: observation, demonstration, instruction, communication, feedback, problem solving and evaluation. An inventory of suitable martial arts clubs with a good reputation for handling children was made. Before John attended the jujutsu training, John's' needs and abilities were discussed with the instructor by telephone. The instructor said that the club had experience of training people with autism disorders. When John arrived at the jujutsu club for the first time, the therapist observed his performance, and strengths and barriers were identified. The following intervention strategies were implemented: (a) practise of step sequences of jujutsu to be able to coordinate arms and legs, (b) doing somersaults backward and forwards, (c) performing controlled falls backwards, (d) learning the vocabulary of jujutsu, the most common instructions in Japanese, and (e) finding the timetable and going alone by bus to and from the jujutsu training. After discussions with John, the therapist wrote down the agreed implementation strategies in John's logbook: what to do, when, how often and with whom. The therapist also documented the planned implementation strategies in the therapist's logbook. John practiced the exercises with the assistance of the therapist at home and at the habilitation centre, and alone at home. John's mother was also instructed in how to assist him with the training at home.

Goal 2

The therapist visited John at home and demonstrated and instructed him how to skate on inlines. Since John already knew how to skate on ice, he got the idea of inline skating relatively rapidly, but he was afraid of falling and wanted to hold on to the therapist. The first times he skated at low speed and was unable to transfer the weight onto one leg, pushing away with the other. He was also unsure of how to stop. The implemented strategies for goal

attainment were: (a) be able to skate with inlines without holding on, (b) to stop without falling, (c) to make turns, (d) to skate at a higher speed, and (e) skate on inlines down a slope without falling. He practiced with the therapist once a week and practiced with his mother between therapist sessions.

John attended group meetings at the habilitation center together with the other child and the therapists once a week for eight weeks. He discussed his positive and negative experiences of the implemented strategies for goal attainment and gave his reflections and some suggestions of how the other child might perform his intervention strategies. During these group meetings, individual practice and planning of the coming week's interventions strategies were carried out. John estimated his performance ability and self-efficacy for goal attainment. He also actively took part in the exercise or social training of how to establish a peer relationship and he played video games.

Evaluation of the intervention process and outcomes

John's goal attainments exceeded the expectations (table 2). He participated regularly once a week in the jujutsu training and was able to go to and from the training by bus on his own (+2). He estimated his performance ability to be 10 and his self -efficacy to 5 (figure 2-4). Concerning the goal attainment of inline skating, he also succeeded very well (+1) and was able to skate 1000 meters without falling and make turns. John estimated his performance ability to 10 and his self-efficacy to 5 (figure 2-4). When asked about the intervention's efficacy and the satisfaction of participation in the intervention, John was in general pleased and said "It *was good to learn martial arts. It was good to learn how to go alone to and from the training. It was fun to try to inline skating*" (table 3). After the intervention John had chosen not to continue to practice jujutsu, and had started to practice tennis once a week. He had also begun to role play detective/spy games with a classmate at school. His mother said that John now seemed to be satisfied with his leisure activities and pleased to have his "own" activity to take part in.

Charlie (pseudonym)

An occupational therapist, implemented the intervention for Charlie.

Charlie (pseudonym) is a male, 14 years old with an autism spectrum disorder. He lives with his family in Uppsala, Sweden. His mother has expressed a wish that her son should develop more social skills.

Measurement of participation patterns and preferences of leisure activities

Charlie responded to the CAPE and PAC questionnaires together with the therapist. He obtained the highest CAPE diversity scores in social activities and performed activities such as, talking on the phone, listening to music, making food, and going to movie together with his family or relatives. He had the highest frequency score in self-improvement activities. Regarding his preferences for activities, the highest preference was found in social activities, followed by self-improvement activities. Charlie identified one goal activity, write a letter and get a pen pal. The goal was formulated using GAS (table 2). At baseline, Charlie's estimation of his performance ability of the goal was 1and his estimated self-efficacy in coping with the goal activity and finally attaining the goal was 3 (figure 5).

Assessment of child-based and environmental facilitators and barriers, and strategies of implementation

Interviews with Charlie and his family were carried out to obtain knowledge of his own and the environmental strengths and hindrances. Charlie was good at expressing what he liked to do, he was faithful and keen on doing good work, and he had a supportive family. The identified barriers for implementation were difficulty in social interaction; he found it hard to know what to say to people, and an awareness of doing or saying the wrong things.

Implementation of strategies for goal attainment

The therapist guided Charlie on how and where he could search to find a website of pen pals. Since Charlie wanted to write letters, rather than sending emails, it took some time to find a suitable site, as most of the websites dealt with email pen pals.
When he finally fined the website, he obtained instruction in to read the personal information about potential pen pals and select at least three persons he wanted to write to.

In the next step, the therapist instructed him how to design and organize a letter. The therapist and Charlie also discussed what were proper things to write about in a letter, how much and what you should reveal about yourself, how to stimulate questions, etc. Since Charlie had chosen a pen pal from Estonia he had to write in English. When he wrote his first letter he got some assistance from his mother and feedback from the therapist. Charlie enjoyed writing and he was instructed to try to write the following two letters without assistance, but with continuous feedback from the therapist.

Charlie also attended group meetings at the habilitation center once a week for eight weeks and discussed positive and negative experiences of the intervention strategies for goal

attainment. Individual practice and planning of the coming week's interventions strategies were carried out, together with estimations of performance ability and self-efficacy for goal attainment. Charlie also participated in social training of how to establish a peer relationship, and he played video games.

Evaluation of the intervention process and outcomes

Charlie attained the goal expectations (table 2). He wrote two letters without assistance, but unfortunately didn't get a reply before the end of the intervention. He estimated his performance ability to be 10 and his self -efficacy to be 5 (figure 5). His estimations of his performance ability and self-efficacy fluctuated during the intervention, mainly because of the disappointment of not getting an answer/reply, but the therapist encouraged him and gave him positive feedback about his writing skills. Charlie was satisfied to have participated in the intervention and he appreciated the group meetings most, since these gave him the opportunity to talk to the therapist (table 5).
